# Hyperprogression of cutaneous T cell lymphoma after anti–PD-1 treatment

**DOI:** 10.1172/jci.insight.164793

**Published:** 2023-02-22

**Authors:** Yumei Gao, Simeng Hu, Ruoyan Li, Shanzhao Jin, Fengjie Liu, Xiangjun Liu, Yingyi Li, Yicen Yan, Weiping Liu, Jifang Gong, Shuxia Yang, Ping Tu, Lin Shen, Fan Bai, Yang Wang

**Affiliations:** 1 Department of Dermatology and Venereology, Peking University First Hospital, Beijing, China.; 2 Beijing Key Laboratory of Molecular Diagnosis on Dermatoses, Beijing, China.; 3 National Clinical Research Center for Skin and Immune Diseases, Beijing, China.; 4 Biomedical Pioneering Innovation Center (BIOPIC), and School of Life Sciences, Peking University, Beijing, China.; 5 Academy for Advanced Interdisciplinary Studies (AAIS), and Peking University–Tsinghua University–National Institute of Biological Sciences Joint Graduate Program (PTN), Peking University, Beijing, China.; 6 Wellcome Sanger Institute, Wellcome Genome Campus, Hinxton, Cambridge, United Kingdom.; 7 BioMap Beijing Intelligence Technology Limited, Block C Information Center Haidian District, Beijing, China.; 8 Department of Lymphoma, Key Laboratory of Carcinogenesis and Translational Research Ministry of Education, and; 9 Department of Gastrointestinal Oncology, Key Laboratory of Carcinogenesis and Translational Research (Ministry of Education/Beijing), Peking University Cancer Hospital and Institute, Beijing, China.; 10 Beijing Advanced Innovation Center for Genomics (ICG), Peking University, Beijing, China.; 11 Center for Translational Cancer Research, Peking University First Hospital, Beijing, China.

**Keywords:** Oncology, Immunotherapy, Lymphomas

## Abstract

**BACKGROUND:**

Immune checkpoint blockade is an emerging treatment for T cell non-Hodgkin’s lymphoma (T-NHL), but some patients with T-NHL have experienced hyperprogression with undetermined mechanisms upon anti–PD-1 therapy.

**METHODS:**

Single-cell RNA-Seq, whole-genome sequencing, whole-exome sequencing, and functional assays were performed on primary malignant T cells from a patient with advanced cutaneous T cell lymphoma who experienced hyperprogression upon anti–PD-1 treatment.

**RESULTS:**

The patient was enrolled in a clinical trial of anti–PD-1 therapy and experienced disease hyperprogression. Single-cell RNA-Seq revealed that PD-1 blockade elicited a remarkable activation and proliferation of the CD4^+^ malignant T cells, which showed functional PD-1 expression and an exhausted status. Further analyses identified somatic amplification of *PRKCQ* in the malignant T cells. *PRKCQ* encodes PKCθ; PKCθ is a key player in the T cell activation/NF-κB pathway. *PRKCQ* amplification led to high expressions of PKCθ and p-PKCθ (T538) on the malignant T cells, resulting in an oncogenic activation of the T cell receptor (TCR) signaling pathway. PD-1 blockade in this patient released this signaling, derepressed the proliferation of malignant T cells, and resulted in disease hyperprogression.

**CONCLUSION:**

Our study provides real-world clinical evidence that PD-1 acts as a tumor suppressor for malignant T cells with oncogenic TCR activation.

**TRIAL REGISTRATION:**

ClinicalTrials.gov (NCT03809767).

**FUNDING:**

The National Natural Science Foundation of China (81922058), the National Science Fund for Distinguished Young Scholars (T2125002), the National Science and Technology Major Project (2019YFC1315702), the National Youth Top-Notch Talent Support Program (283812), and the Peking University Clinical Medicine plus X Youth Project (PKU2019LCXQ012) supported this work.

## Graphical abstract

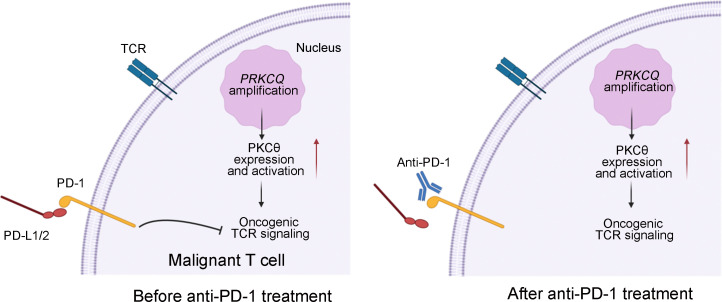

## Introduction

Cutaneous T cell lymphomas (CTCLs) compose a group of T cell non-Hodgkin’s lymphomas (T-NHLs) of skin-homing T cells (1). Mycosis Fungoides (MF) and Sézary syndrome (SS), accounting for 65% of all CTCLs, are characterized by the clonal expansion of skin resident CD4^+^ T cells (2). In patients with advanced stage (IIB–IV) MF and SS, the responses to systemic multiagent chemotherapies are usually short-lived, and the prognosis of these patients is generally poor (3); therefore, new treatment modalities for these types of CTCLs are urgently needed. In the last decade, immune checkpoint inhibitor therapies, including anti–PD-1 and anti–PD-L1 antibodies, have led to revolutionary changes in the management of multiple solid tumors. Clinical trials of anti–PD-1 therapy in patients with MF/SS have reported an overall response rate of 15%–38% (4, 5), suggesting that this modality could hold promise as a treatment for patients with CTCLs. Nevertheless, 25%–31% of advanced patients with MF/SS experienced progression after anti–PD-1 therapy (4, 5). Moreover, 2 clinical trials of anti–PD-1 therapy in patients with peripheral T cell lymphomas (PTCL), including MF and SS, were halted due to a high progression rate (6) or lack of efficacy (7). It is of great necessity to address the mechanisms underlying the progression of T cell lymphoma upon anti–PD-1 therapy to guide precise and safe immunotherapy.

## Results

### A patient with MF experienced hyperprogression upon anti–PD-1 therapy.

**Figure 1:**
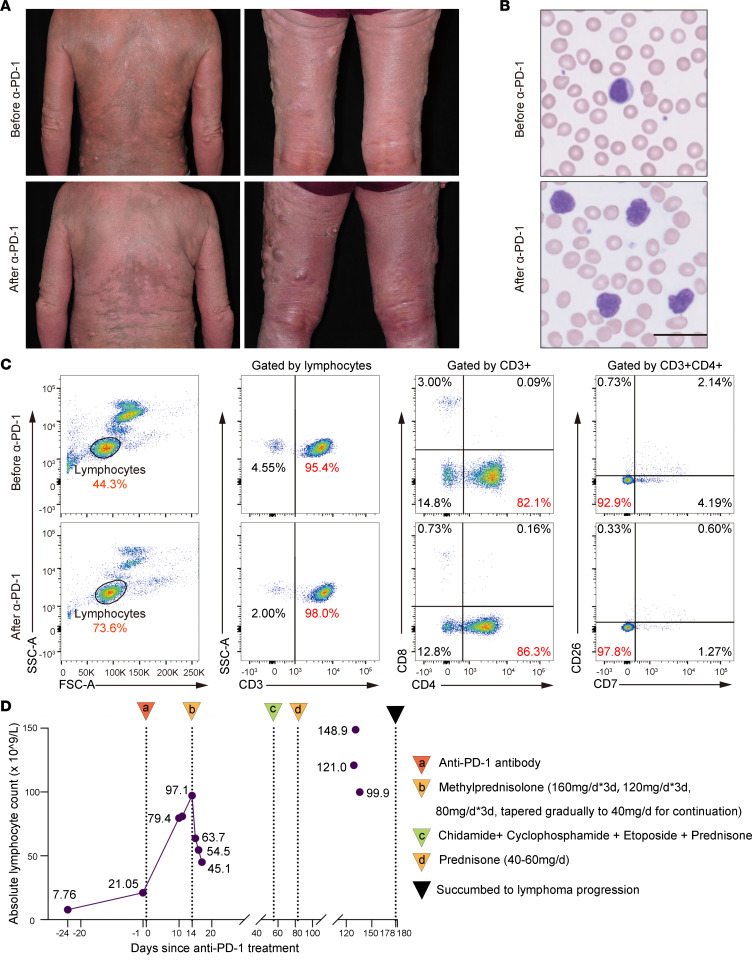
A patient with MF experienced hyperprogression upon anti–PD-1 therapy. (**A**) Clinical images of the patient before and after anti–PD-1 treatment. (**B**) Atypical lymphocytes in peripheral blood smear before and after anti–PD-1 treatment. Scale bar: 25 μm. (**C**) Flow cytometry analysis of peripheral blood from the patient before and after anti–PD-1 treatment. The percentages of lymphocytes are indicated. (**D**) Clinical course and absolute lymphocyte counts in peripheral blood of the patient before and after anti–PD-1 treatment. Arrowheads labeled a–d indicate the treatment administration. *3d, 3 days.

A 52-year-old man who had a 13-year history of MF presented to our clinic with generalized erythroderma and multiple tumors in October, 2018 (Figure 1A). Psoralen-UVA photochemotherapy, s.c. IFN-α, and systemic chemotherapy with the cyclophosphamide, doxorubicin, vincristine, etoposide, and prednisone (CHOEP) regimen had failed to impede the progression. Skin biopsy showed tumor stage MF with large cell transformation. A CT scan of the chest, abdomen, and pelvis revealed enlarged superficial and mediastinal lymph nodes, and a lymph node biopsy showed malignant infiltration classified as N2. A peripheral blood smear showed multiple large, atypical lymphoid cells with cerebriform nuclei (Figure 1B). The absolute lymphocyte count in peripheral blood was 7.76 × 10^9^/L. Flow cytometric analysis of peripheral blood showed the majority of lymphocytes were CD3^+^ cells (95.4%) and a CD4/CD8 ratio of 27.4:1 (range, 0.5–2:1), with the majority of CD4^+^ cells (92.9%) showing a CD7^–^CD26^–^ phenotype, indicating the peripheral blood involvement of lymphoma (Figure 1C). The HTLV-1–derived gene *pol* was negative (8), excluding adult T cell leukemia/lymphoma (ATLL) (Supplemental Figure 1; supplemental material available online with this article; https://doi.org/10.1172/jci.insight.164793DS1). The patient was diagnosed as advanced MF (stage IVA1) and was enrolled in a phase IA clinical trial of a humanized IgG4 anti–PD-1 monoclonal antibody (9) in advanced lymphomas and solid tumors (NCT03809767). He was given an initial dose of 60 mg monotherapy, in a plan for dose escalation. His skin lesions progressed shortly after the initial dose, with skin tumors enlarging and ulcerating as well as aggravated skin itching and swelling (Figure 1A). A substantial increase of atypical lymphocytes in blood smear was observed 12 days after the treatment (Figure 1B). The absolute lymphocyte count in peripheral blood rose to 79.4 × 10^9^/L (10 days after anti–PD-1 treatment), and the CD4/CD8 ratio rose to 118.2:1 with an increased proportion of CD4^+^CD7^–^CD26^–^ malignant T cells (97.8%) (Figure 1C), demonstrating the occurrence of hyperprogression (defined as time-to-treatment failure of equal or less than 1 month of therapy with symptomatic progression; ref. 6). Anti–PD-1 therapy was discontinued, and high-dose methylprednisolone was initiated to control the disease progression (Figure 1D). The patient’s disease progressed rapidly afterward, and he succumbed to the disease progression 6 months later.

### Anti–PD-1 treatment promoted the activation and proliferation of malignant T cells.

**Figure 2:**
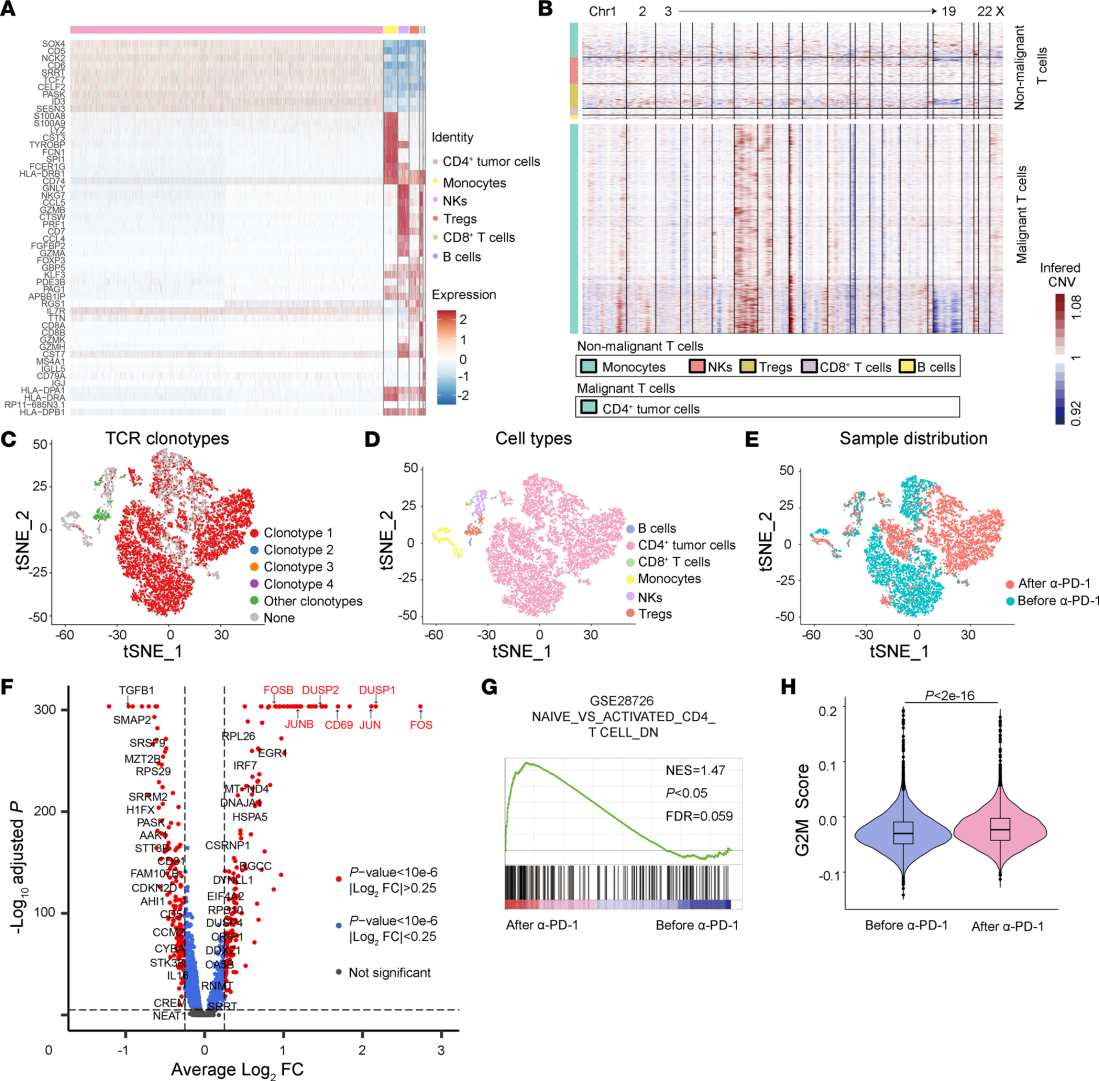
Anti–PD-1 treatment promoted the activation and proliferation of malignant T cells. (**A**) Expression levels of the top 10 signature genes in each cell type from the 10x Genomics data set. Expression is indicated as the *Z* score–normalized log_2_ level (count+1). (**B**) Large-scale CNVs of single cells from all samples. CNVs were inferred from the 10x Genomics data set. (**C**) The T-SNE plot shows all cells from the 10x Genomics data set after clustering. Each color represents a distinct TCR clonotype. (**D**) The T-SNE plot shows cell types of all PBMCs before and after anti–PD-1 treatment, with cells colored by cell types. (**E**) The T-SNE plot shows the sample distribution all PBMCs before and after anti–PD-1 treatment, with cells colored by sample ID. (**F**) Volcano plot of differentially expressed genes in all CD4^+^ malignant T cells after anti–PD-1 treatment versus before anti–PD-1 treatment. (**G**) GSEA of differentially expressed genes detected by scRNA-Seq in all malignant T cells after anti–PD-1 treatment versus before anti–PD-1 treatment. NES, normalized enrichment score. (**H**) G2-M score of malignant T cells of the patient before and after anti–PD-1 treatment from the 10x Genomics data set.

To dissect the response of malignant T cells and nonmalignant cells to anti–PD-1 treatment, PBMCs obtained from the patient before and after PD-1 blockade were subjected to single-cell RNA-Seq (scRNA-Seq). Six major cell types were identified based on the expression of canonical marker genes, inferred copy number variations (CNVs) (10), and matched T cell receptor α (TCRα) and TCRβ clonotypes at the single-cell level (11); cell types included CD4^+^ malignant T cells, monocytes, NK cells, Tregs, CD8^+^ T cells, and B cells (Figure 2, A–D, and Supplemental Figure 2). T-distributed stochastic neighbor embedding (T-SNE) analysis of scRNA-Seq data showed drastic alteration in the gene expression profile of CD4^+^ malignant T cells after anti–PD-1 treatment, in contrast to the relatively unchanged gene expression profile of CD8^+^ T cells (Figure 2, D and E). The most upregulated genes in malignant T cells after anti–PD-1 treatment included *FOS*, *DUSP1*, *JUN*, *CD69*, *DUSP2*, *JUNB*, and *FOSB* (Figure 2F and Supplemental Table 1), which are gene signatures for TCR signaling and T cell activation (12, 13). CD69, an established marker for T cell activation (14) and a critical target suppressed by PD-1/PD-L1 signaling (15, 16), was highly upregulated in malignant T cells upon anti–PD-1 treatment (Figure 2F). Gene-set enrichment analysis (GSEA) further confirmed the enrichment of genes involved in T cell activation pathways (17) in malignant cells after treatment (Figure 2G). The G2-M score of malignant T cells significantly increased after anti–PD-1 treatment (Figure 2H), indicating augmented proliferation of malignant T cells by anti–PD-1 treatment. These results collectively suggest that the low-dose anti–PD-1 treatment elicited the activation and proliferation of CD4^+^ malignant T cells in the patient.

### PD-1 signaling inhibited proliferation of malignant T cells.

**Figure 3:**
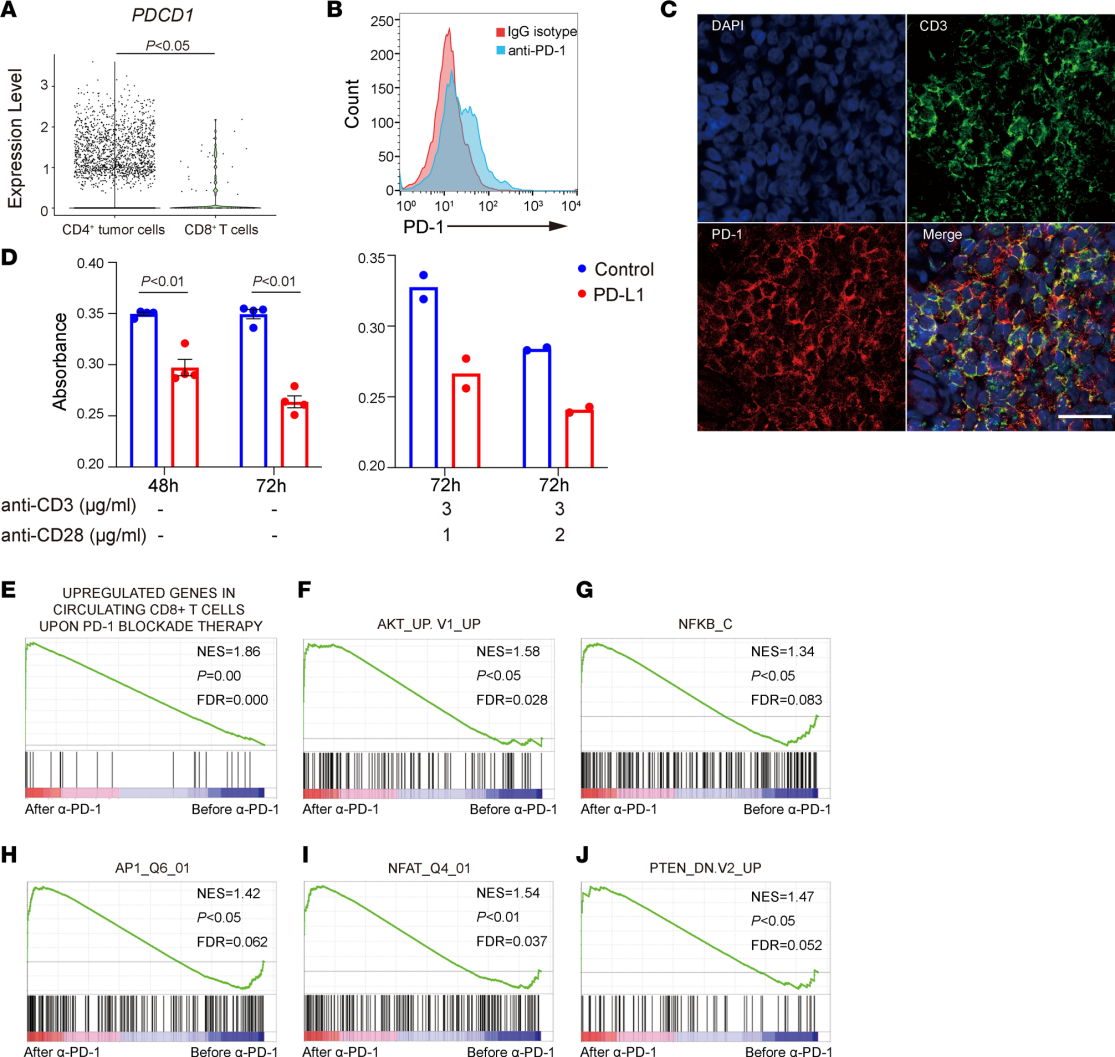
PD-1 signaling inhibited proliferation of malignant T cells. (**A**) The violin plot shows the expression level of *PDCD1* in CD4^+^ tumor T cells and CD8^+^ T cells at the single-cell level in PBMCs before anti–PD-1 treatment. The results were analyzed with the Wilcoxon ranked-sum test with continuity correction. (**B**) Expression of PD-1 detected by flow cytometry on PBMCs before anti–PD-1 treatment. Cells were stained with an anti–PD-1 antibody or its isotype IgG. (**C**) Immunofluorescence staining of formalin-fixed, paraffin-embedded skin tumor tissue for PD-1 and CD3 before anti–PD-1 treatment. Original magnification, 400×. Scale bar: 25 μm. (**D**) Proliferation of PBMCs treated with human PD-L1 Fc chimeric protein or human IgG1Fc before anti–PD-1 therapy. Two-tailed Student’s *t* test was utilized for the left panel. The results are shown as the mean ± SEM of data from 2 independent experiments for the left panel. The right panel shows data from 1 experiment. (**E**–**J**) GSEA of differentially expressed genes detected by scRNA-Seq in all malignant T cells after anti–PD-1 treatment versus before anti–PD-1 treatment. NES, normalized enrichment score.

To explore the mechanisms underlying malignant T cell activation and tumor progression upon PD-1 blockade, we evaluated PD-1 expression in lymphoma tissue samples. scRNA-Seq and flow cytometric analysis of PBMCs isolated from the patient before treatment showed PD-1 expression on malignant T cells (Figure 3, A and B), and this was validated by immunofluorescence staining of skin tumor samples (Figure 3C). The *PDCD1* expression level of malignant T cells was much higher than that of CD8^+^ reactive T cells (Figure 3A), and this might partially explain the relatively unchanged gene expression profile of CD8^+^ reactive T cells after treatment, given that the patient received only a low dose of anti–PD-1 therapy. The malignant T cells express other coinhibitory receptors like *TIGIT*, *LAG3*, *CTLA4*, exhaustion marker *ENTPD1*, and transcription factor *TOX*, but they rarely express exhaustion marker *CXCL13*; coinhibitory receptors like *LAYN* and *HAVCR2*; or transcription factor *TOX2* (Supplemental Figure 3, A–I), suggesting that the malignant T cells in our patient may fit the “moderately exhausted CTCL” defined by Park et al., which retained the ability to proliferative to TCR stimuli ex vivo (18). Expression levels of *TIGIT*, *PDCD1*, and *TOX* were downregulated, and expression of *CTLA4* was upregulated with statistical significance, but expression levels of other exhaustion related markers were not significantly changed upon anti–PD-1 treatment (Supplemental Figure 3, A–J). Recombinant PD-L1, a ligand for PD-1, inhibited the proliferation of the patient’s primary malignant T cells before anti–PD-1 treatment among PBMCs cultured ex vivo, with or without TCR activation by anti-CD3/anti-CD28 antibodies (Figure 3D). These results demonstrate the tumor-suppressive role of PD-1 signaling in malignant T cells.

Previous studies have reported that the PD-1 signaling pathway functions by inhibiting the PI3K/AKT and PKCθ/NF-κB signaling pathways, inhibiting activation of transcription factors like activator protein 1 (AP-1) and nuclear factor of activated T cells (NFAT) and activating phosphatase and tensin homolog deleted on chromosome 10 (PTEN) in T cells (19, 20). These pathways are also important for driving oncogene-transformed T cell proliferation and survival in T cell lymphomas (21–23). Accordingly, in this case, genes upregulated after anti–PD-1 treatment in malignant T cells were found to be enriched with genes from a gene set upregulated by PD-1 blockade in peripheral blood T cells (Figure 3E) (24), as well as with genes upregulated by AKT (Figure 3F), NF-κB (Figure 3G), AP-1 (Figure 3H), and NFAT (Figure 3I) and with genes downregulated by PTEN (Figure 3J). These results show that the anti–PD-1 therapy in this patient blocked the PD-1 signaling pathway on malignant T cells and promoted the lymphoma hyperprogression of this patient.

### Genomic PRKCQ amplification contributed to the constitutive activation of TCR signaling in malignant T cells.

Previous studies from our group and others reported PD-1 expression on malignant T cells in 19%–39.4% of CTCL cases (25, 26). Clinical trials of anti–PD-1 antibodies revealed that the PD-1 expression status or deletion was unrelated to anti–PD-1 therapy outcomes in CTCL patients (4), implying that factors other than PD-1 expression determined the tumor-suppressive activity of the PD-1 signaling pathway in CTCLs. PD-1 was shown to act as a haploinsufficient suppressor of T cell lymphomagenesis in a mouse model with constitutive TCR activation induced by *ITK-SYK* fusion in T cells, in which PD-1 counteracts oncogenic TCR signaling in CD4^+^ T cells (19). This study showed that the inhibitory function of PD-1 signaling requires the activation of TCR signaling, which was also suggested by previous observations (27–29). In CTCLs, recurrent mutations in components of the T cell activation/NF-κB pathway have been identified frequently and have been regarded as putative driver mutations (30–32).

**Figure 4:**
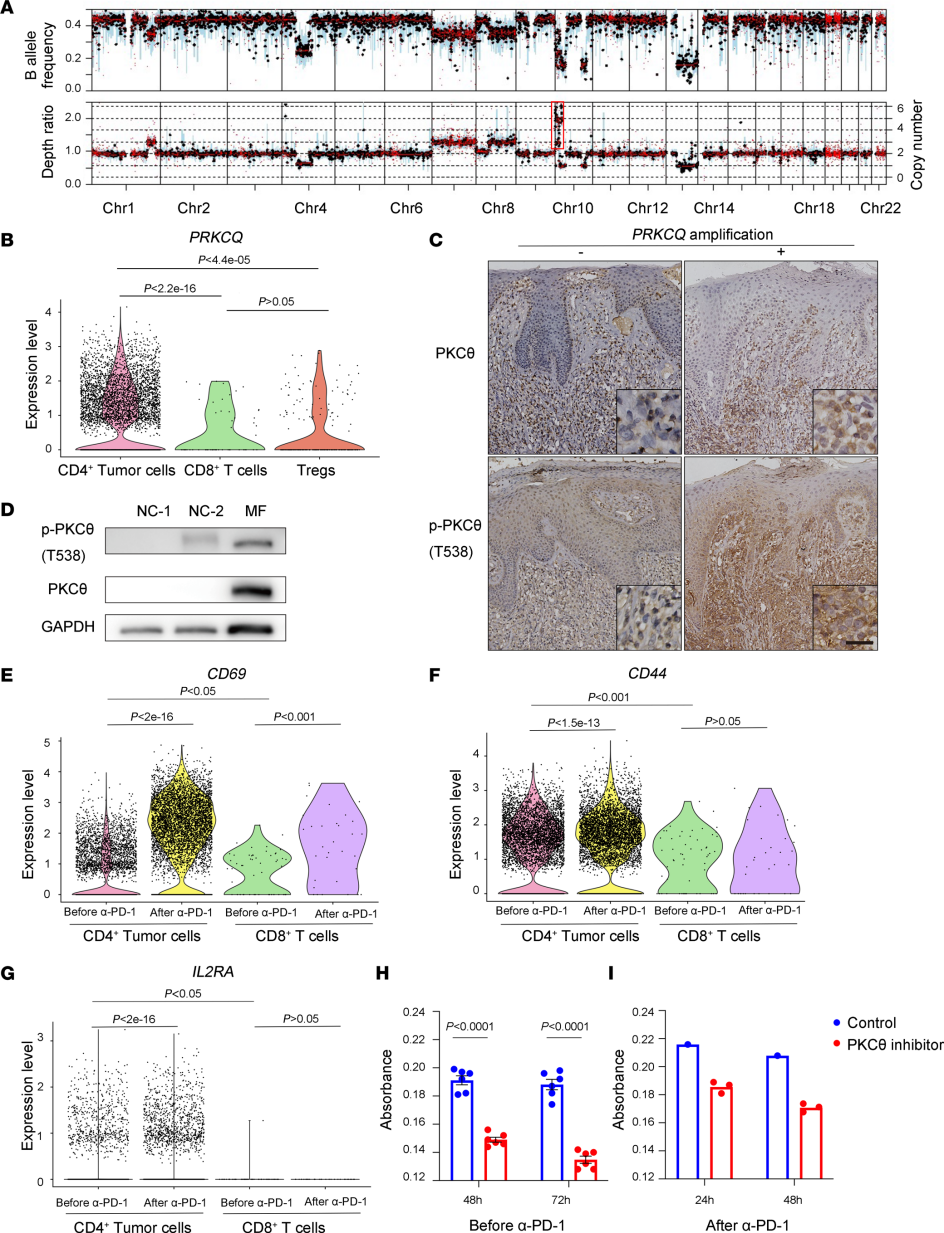
Genomic *PRKCQ* amplification contributes to the constitutive activation of TCR signaling in malignant T cells. (**A**) Chromatin aberration detected by WGS of genomic DNA extracted from PBMCs before anti–PD-1 treatment compared with granulocyte genomic DNA in this patient. (**B**) The violin plot shows the expression level of *PRKCQ* in CD4^+^ tumor T cells, CD8^+^ T cells, and Tregs at the single-cell level in PBMCs before anti–PD-1 treatment. The data were analyzed using the Wilcoxon ranked-sum test with continuity correction. (**C**) IHC staining of PKCθ and p-PKCθ (T538) on skin tumors. The images in the left panel are representative of the results obtained using skin tumors from 5 patients with MF without *PRKCQ* amplification. The images in the right panel are results obtained using tumor sample obtained before the patient received anti–PD-1 treatment. Scale bar: 25 μm. (**D**) Western blot results showing PKCθ and p-PKCθ (T538) expression in the cell lysate of PBMCs after anti–PD-1 treatment from the patient. Cell lysates of PBMCs from 2 healthy donors served as the normal control (NC). (**E**–**G**) The violin plots show the expression levels of markers of T cell activation on CD4^+^ tumor T cells and CD8^+^ T cells before and after anti–PD-1 treatment at the single-cell level in PBMCs. The data were analyzed using the Wilcoxon ranked-sum test with continuity correction. (**H** and **I**) Proliferation of PBMCs that were obtained from the patient before (**H**) and after (**I**) anti–PD-1 therapy and treated with or without Compound 20 (5 μM), a PKCθ-specific inhibitor. Student’s *t* test was utilized. The results are shown as the mean ± SEM of data from 3 independent experiments for (**H**). Data from one experiment are shown for **I**.

To explore the mechanism underlying the tumor-suppressive activity of PD-1 signaling in this patient and assess the presence of oncogenic TCR activation, we performed both whole-exome sequencing (WES) and whole-genome sequencing (WGS) on the patient’s PBMCs before anti–PD-1 treatment. Data from WES identified 52 nonsynonymous SNVs, 4 nonsense SNVs, and 1 frame shift deletion (Supplemental Table 3). Among them, *TRRAP* was a putative oncogene and *CREBBP* was a putative tumor suppressor in CTCL, as reported by Park et al. (18). However, both SNVs showed low allele frequencies (18% for *TRRAP* and 19% for *CREBBP*), and neither of the mutations were key factors in TCR signaling or PD-1 signaling. With WGS, 4 fusion genes were detected; the unique reads of these fusion genes in WGS are no more than 6, indicating that these fusion genes were present in few malignant T cells and that it is unlikely that they play important role in the lymphoma hyperprogression upon anti–PD-1 treatment in this patient (Supplemental Table 4). According to putative driver genes in TCR signaling identified by Park et al. (18), a mild *CARD11* amplification (copy number = 3), a mild *PTPRN2* amplification (copy number = 3), a mono-allelic *NFKB2* deletion (copy number = 1), and a mono-allelic *PDCD1* deletion (copy number = 1) were also detected (Supplemental Tables 2 and 5). *CARD11* and *PTPRN2* are identified as oncogenes of CTCL by Park et al. (18). However, the expression levels of these 2 genes were not significantly altered on malignant T cells compared with their counterpart CD8^+^ reactive T cells, according to our scRNA-Seq data (Supplemental Figure 4, A and B). Of note, a drastic chromosome amplification on chr10q was identified by WGS (Figure 4A), which resulted in a copy number gain of 6 times for gene *PRKCQ* (Supplemental Tables 2 and 5). *PRKCQ* encodes PKCθ, a critical effector molecule of TCR and CD28 signaling and a key player in the T cell activation/NF-κB pathway, which promotes the activation, proliferation, and survival of T cells (33). Our scRNA-Seq data also show significant upregulation of *PRKCQ* expression on malignant T cells in comparison with reactive T cells (Figure 4B). PKCθ is activated by phosphorylation of T538 (34). We found high expression levels of PKCθ and p-PKCθ (T538) on malignant T cells in the patient’s skin tissue samples in comparison with those found on skin tumors from patients with MF without *PRKCQ* amplification (Figure 4C). Expressions of PKCθ and p-PKCθ (T538) on malignant T cells in the patient’s PBMCs were also detected (Figure 4D).

To explore whether *PRKCQ* amplification activated constitutional TCR signaling and whether this activation was further released by anti–PD-1 treatment, we analyzed the expressions of T cell activation markers, including *CD69*, *CD44*, and *IL2RA*, which are PKCθ downstream effectors during T cell activation (35). Compared with *PRKCQ* WT CD8^+^ T cells, *CD69*, *CD44*, and *IL2RA* were significantly upregulated on malignant CD4^+^ T cells, and the expressions of these genes further showed a more remarkable increase after anti–PD-1 treatment (Figure 4, E–G). Moreover, the molecular pathways in which the upregulated genes on posttreatment malignant T cells — including NF-κB, AP-1, and NFAT — are enriched were also critical PKCθ downstream pathways (36–38) (Figure 3, G–I). Next, we treated the patient’s primary malignant T cells in PBMCs isolated before and after anti–PD-1 treatment with Compound 20 (Selleck), a specific PKCθ inhibitor (39). The proliferations of malignant T cells, both before and after anti–PD-1 treatment, were inhibited by Compound 20 ex vivo (Figure 4, H and I), demonstrating the activation status and oncogenic consequences of PKCθ signaling in primary malignant T cells. Collectively, these data demonstrate that overexpression and activation of PKCθ caused by *PRKCQ* amplification resulted in oncogenic activation of the TCR signaling pathway in the patient, setting the stage for the tumor-suppressive effect of PD-1 signaling in malignant T cells.

## Discussion

**Figure 5:**
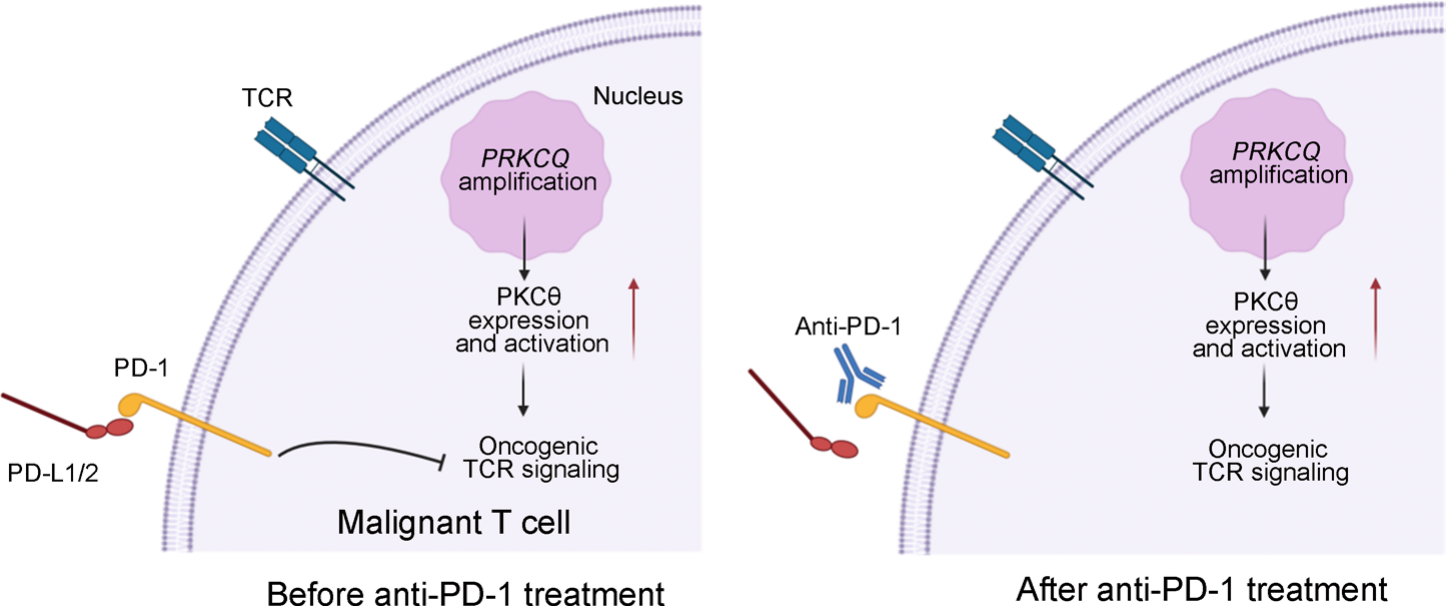
Schematic model of mechanism underlying hyperprogression after anti–PD-1 treatment in the cutaneous T cell lymphoma patient. PD-1 acts as a tumor suppressor for malignant T cells with oncogenic TCR activation caused by *PRKCQ* amplification; PD-1 blockade treatment released oncogenic TCR signaling and led to the proliferation and activation of malignant T cells.

The mechanism underlying the progression of T cell lymphoma after anti–PD-1 immunotherapy remains largely unknown. Our study reveals that PD-1 exerted a tumor suppressive role on malignant T cells with constitutive TCR activation; PD-1 blockade therapy ameliorated the inhibitory role of PD-1 signaling on malignant T cells, thereby accelerating the progression of CTCL (Figure 5).

Physiologically, PD-1 is upregulated upon TCR engagement, and PD-1 signaling inhibits the proliferation of T cells with TCR signaling activation (40), whereas the function of PD-1 on malignant T cells has not been fully elucidated. Studies have found recurrent genomic loss of the *PDCD1* gene in T-NHLs (30), and in patients with stage II–III MF, deletion of *PDCD1* is a marker for dismal prognosis (18). A previous study demonstrated that malignant T cells of SS patients express PD-1 and that PD-1 signaling could inhibit proliferation of primary malignant T cells activated by PMA/ionomycin in vitro (29). In an engineered T-NHL mouse model with forced *ITK-SYK* expression in CD4^+^ T cells, *Pdcd1* deletion resulted in complete malignant transformation of T cells, and blockade of PD-1 signaling by the administration of anti–PD-1 or anti-PD-L1 antibodies accelerated the progression of lymphoma in vivo, suggesting that PD-1 functions as a powerful suppressor of lymphomagenesis under certain conditions and models (19).

Our findings from single-cell transcriptome analyses before and after treatment suggest that the tumor-suppressive mechanisms of PD-1 overlap with physiological PD-1 signaling, consistent with findings from the *ITK-SYK* mouse model, which showed that anti–PD-1 antibody treatment facilitated lymphoma progression by releasing the PI3K/AKT and PKCθ/NF-κB pathways and inhibiting the tumor-suppressive PTEN pathway in malignant T cells (19). Physiological PD-1 signaling reduces the phosphorylation of ZAP70/CD3ζ and inhibits downstream signaling to PKCθ (41). The PKCθ/NF-κB pathway is as a critical promoter of T cell lymphomagenesis. A recent study revealed that PKCθ promoted CTCL development and progression in a chicken embryo xenograft (42), supporting the idea that *PRKCQ* functions as a potent oncogene in CTCLs. In this case, *PRKCQ* amplification led to PKCθ overexpression and constitutional TCR activation in malignant T cells. Although harboring a monoallelic *PDCD1* deletion, these cells expressed functional PD-1 and other exhaustion markers, demonstrating a moderately exhausted phenotype, while the PD-1 blockade released proximal TCR signals and led to the disinhibition of oncogenic PKCθ/NF-κB signaling, resulting in lymphoma progression and deterioration of the patient.

Studies based on high-throughput sequencing in the past decade have revealed that oncogenic genetic aberrations in components of the T cell activation/NF-κB pathway are hallmarks of T cell lymphomas (30, 31). Activating mutations in the NF-κB pathway (for example, in *PLCG1*, *CARD11*, and *TNFRSF1B*) have been identified in CTCLs. *PRKCQ* amplification has been found in 20%–53% of advanced-stage CTCL patients (43–45). Our data, in combination with the results of previous studies, suggest that the tumor-suppressive function of PD-1 signaling in T cell lymphoma may be not restricted to *PRKCQ* amplification and may be applicable in other patients with oncogenic TCR activation, although the intracellular pathways that mediate PD-1 function in lymphoma cells are inadequately defined and it is still unknown whether PD-1 expression on malignant T cells is a direct result of T cell oncogenesis. Therefore, this case report suggests that genetic evaluation of TCR pathway molecules and assessment of PD-1 expression on malignant T cells may be required as a method for identifying patients with T cell lymphoma who might experience progression upon PD-1 blockade treatment. Furthermore, particular attention should be paid to the potential for secondary T cell lymphomas in patients treated with PD-1 checkpoint inhibitors.

In addition, lymphoma hyperprogression should be distinguished from skin flare reaction after anti–PD-1 treatment. In the phase II trial of pembrolizumab in CTCL, 53% of patients with SS experienced marked worsening of erythema and pruritus during treatment (4). This skin flare reaction eventually subsided with only supportive care and without discontinuation of anti–PD-1 treatment (4). It showed different clinical courses with the hyperprogression in our patient. Hence, a thorough clinical workup, including flow cytometry of peripheral blood or imaging, should be performed to assess the patients’ response during immunotherapy.

Our work has several limitations. The single-case design of this study makes it challenging to draw an exclusive link between *PRKCQ* amplification and hyperprogression upon anti–PD-1 treatment. Other genetic abnormalities may also be implicated in the hyperprogression upon anti–PD-1 treatment in our patient. The conclusion of this study should be further validated in additional T cell lymphoma patients in future studies.

Taken together, the results of our study provide real-world clinical evidence that PD-1 acts as a tumor suppressor for malignant T cells with oncogenic TCR activation. These results highlight the importance of tumor genomic profiling in improving the clinical benefit of anti–PD-1/PD-L1 regimens in patients with T cell lymphomas in the future.

## Methods

### Peripheral blood flow cytometric analysis.

Peripheral blood was obtained 24 days before and 12 days after the initiation of anti–PD-1 treatment. Flow cytometric analysis with antibodies recognizing CD3/CD4/CD8/CD7/CD26 (catalogs 663490, 663493, 341051, 663502, and 340426, respectively; BD Biosciences) was performed on a BD FACSCanto Clinical Flow Cytometry System. Flow cytometric analysis with antibodies recognizing PD-1 (BioLegend, 329907) or mouse IgG1, κ Isotype control (BioLegend, 400122) on PBMCs before anti–PD-1 treatment was performed on a BD FACSCalibur Flow Cytometry System.

### scRNA-Seq and paired single-cell TCR-Seq.

PBMCs were resuscitated and subjected to scRNA-Seq on the 10x Genomics platform. The 10x Genomics single-cell 5′ reagent kit was coupled with TCR V(D)J analyses to profile TCR clonotypes at a single-cell resolution. scRNA-Seq reads were aligned to the hg19 human reference genome using “CellRanger count” (v3.0.2), and scRNA-TCR data were aligned to the vdj-GRCh38 reference genome.

### WGS and WES.

Genomic DNA was obtained from PBMCs before anti–PD-1 treatment. It was subjected to WGS on the Illumina HiSeq X Ten platform with the sequencing depth set to 50× (Illumina), and it was subjected to WES on the Illumina Novaseq 6000 platform with the sequencing depth set to 200× (Illumina). Genomic DNA from peripheral blood granulocytes was used as the germline control.

### Ex vivo cell assay.

PBMCs were resuscitated and cultured in RPMI-1640 medium (Sigma-Aldrich) with 10% FBS, 100 U/mL penicillin, and 0.1 mg/mL streptomycin. For PD-L1 treatment, 96-well round-bottom plates were precoated with human IgG1 Fc (10 μg/mL, R&D Systems, 110-HG-100) as the control or human PD-L1 Fc Chimera Protein (10 μg/mL, R&D Systems, 156-B7-100) overnight at 4°C. The cells were seeded in duplicate plates at a density of 1 × 10^5^ cells/100 μL medium. For PKCθ inhibitor treatment, PBMCs were treated with 5 μmol/L Compound 20 (Selleck, S6577) or vehicle (DMSO) for 30 minutes, washed with PBS, and seeded in duplicate plates at a density of 1 × 10^5^ cells/100 μL medium. After the cells were cultured for indicated time points in the figure legends, absorbance at a wavelength of 490 nm was measured with the CellTiter 96 AQueous One Solution Cell Proliferation Assay (Promega, 3580).

### IHC and immunofluorescence staining.

Slides of skin lesions before anti–PD-1 treatment were subjected to IHC or immunofluorescence staining with antibodies against PKCθ (Cell Signaling Technology, 13643), p-PKCθ (BD Biosciences, 612735), CD3 (Abcam, ab699), and PD-1 (Abcam, 137132).

### Western blotting.

Whole-cell lysates of patient PBMCs after anti–PD-1 treatment or PBMCs from healthy donors were separated by SDS-polyacrylamide electrophoresis and then probed with antibodies against p-PKCθ (BD Biosciences, 612735), PKCθ (Cell Signaling Technology, 13643), and GAPDH (ZSGB-BIO, TA-08).

### Data availability.

The raw data from the scRNA-Seq, TCR-Seq, WES, and WGS have been deposited in the Genome Sequence Archive for human (GSA-Human) under accession no. HRA000163.

### Statistics.

Methods of statistical analysis were indicated in figure legends. Statistical significance was accepted at *P* less than 0.05. In this study, R (version 4.0.3) and GraphPad Prism (version 9.4.1) software were used to conduct statistical analysis. Wilcoxon ranked-sum test with continuity correction and 2-tailed Student’s *t* test were used

### Study approval.

The study protocol was approved by the Ethics Committee of Peking University First Hospital. The patient provided written informed consent indicating his understanding that the peripheral blood and skin biopsies were obtained for disease evaluation and scientific research. The patient also provided written informed consent for the use of the photographs, and the record of informed consent has been retained.

## Author contributions

YG, FB, and YW designed the study. YG, YW, FL, YL, YY, PT, SY, WL, JG, and LS collected and analyzed the clinical data of the patient. YG and SJ conducted the experiments. YG, SH, RL, SJ, XL, FB, and YW acquired and analyzed the experimental data. YG, SH, FB, and YW wrote the manuscript with the contribution from all coauthors. FB and YW supervised the project. Order of co–first authors was decided based on the timeline of their contributions.

## Supplementary Material

Supplemental data

ICMJE disclosure forms

Supplemental table 1

Supplemental table 2

Supplemental table 3

Supplemental table 4

Supplemental table 5

## Version 1 01/17/2023

In-Press Preview

## Version 2 02/22/2023

Electronic publication
