# Supplementary material for: Hyperprogression of cutaneous T cell lymphoma after anti–PD-1 treatment
Source: JCI Insight. 2023 Feb 22;8(4):e164793. doi: 10.1172/jci.insight.164793 (PMC9977500; doi:10.1172/jci.insight.164793)
Supplement: Supplemental data [file jciinsight-8-164793-s094.pdf]

# Supplemental Materials

## Additional methodological details

### Polymerase chain reaction (PCR) and gel electrophoresis

PCR was performed using specific primers amplifying a 189 bp fragment of the *pol* gene of HTLV-1 on genomic DNA from primary malignant T cells in peripheral blood and CTCL cell lines (1). The PCR product was separated in 2% agarose gel to identify positive samples.

## Supplemental figures

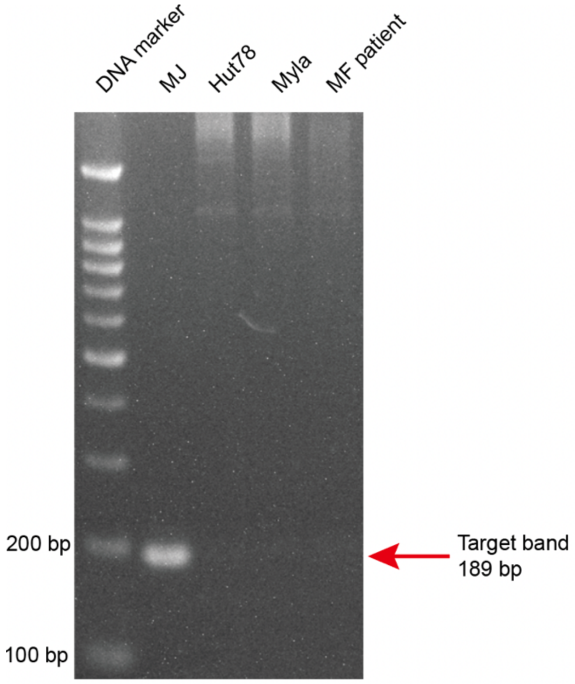


**Supplemental Figure 1. Image of gel electrophoresis after HTLV-1 PCR.**

Image of gel electrophoresis after PCR targeting HTLV-1 using genomic DNA from the indicated cell lines and PBMCs from the patient. MJ cells, which originated from a HTLV-1-positive MF patient, served as the positive control; Hut78 and Myla cells, which originated from MF patients without HTLV-1 infection, served as the negative control.

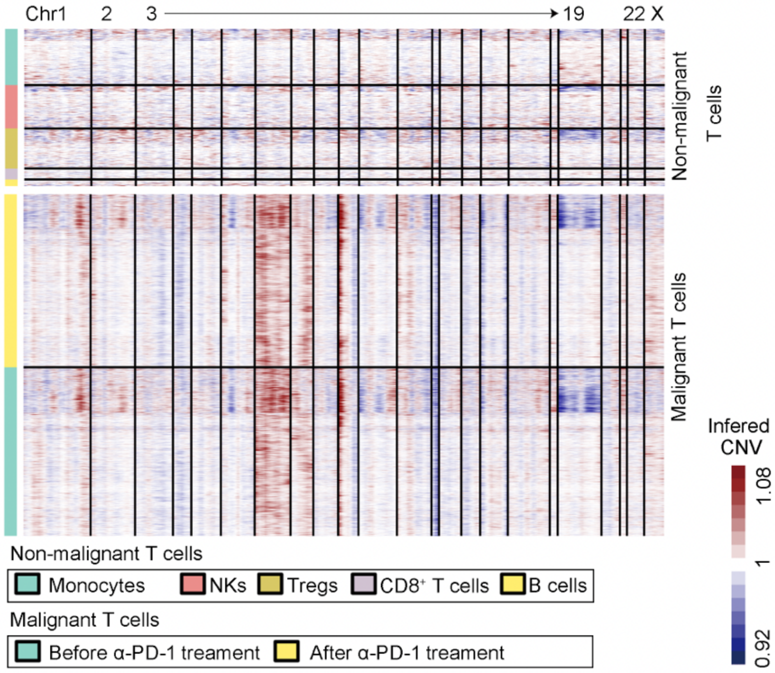


**Supplemental Figure 2. Large-scale CNVs of malignant T cells before and after anti-PD-1 treatment.**

Large-scale CNVs of single cells from all samples. CNVs were inferred from the 10x Genomics dataset.

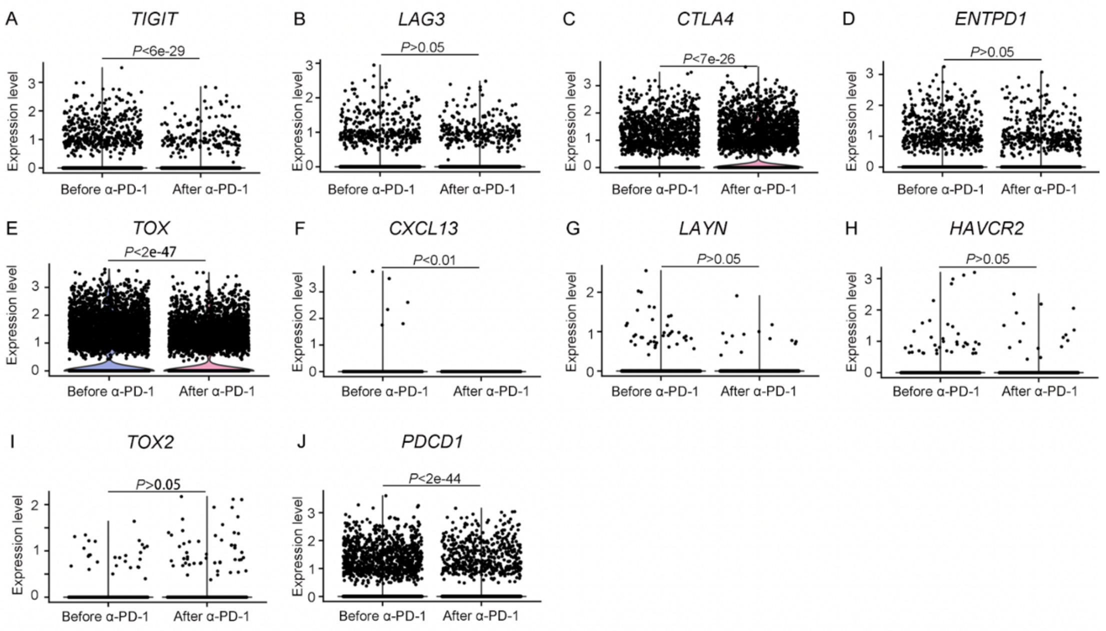


**Supplemental Figure 3. T cell exhaustion-related markers on CD4+ tumor T cells.**

(**A-J**) The violin plots show the expression level of T cell exhaustion-related markers on CD4+ tumor T cells at the single-cell level in PBMCs before and after anti-PD-1 treatment. The results were analyzed with the *Wilcoxon rank sum test* with continuity correction.

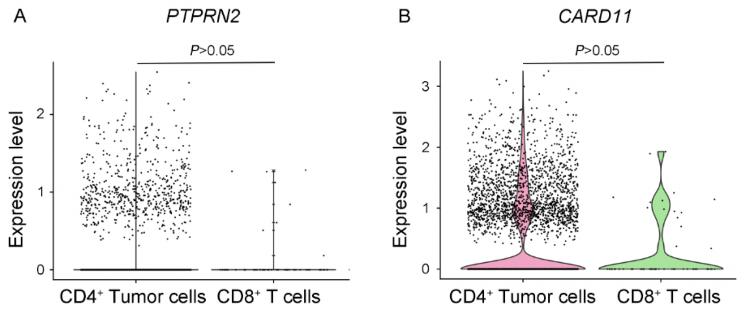


**Supplemental Figure 4. Expression levels of *PTPRN2* and *CARD11* in CD4+ tumor T cells and CD8+ T cells**

The violin plots show the expression level of *PTPRN2* (**A**) and *CARD11* (**B**) in CD4+ tumor T cells and CD8+ T cells at the single-cell level in PBMCs before anti-PD-1 treatment. The results were analyzed with the *Wilcoxon rank sum test* with continuity correction.

## Supplemental references

1. Balcázar N, et al. Sequence and phylogenetic analysis of human T cell lymphotropic virus type 1 from Tumaco, Colombia. *Memorias do Instituto Oswaldo Cruz*. 2003;98(5):641-648.
